# Selection of opioids for cancer-related pain using a biomarker: a randomized, multi-institutional, open-label trial (RELIEF study)

**DOI:** 10.1186/s12885-017-3664-z

**Published:** 2017-10-06

**Authors:** Hiromichi Matsuoka, Junji Tsurutani, Yasutaka Chiba, Yoshihiko Fujita, Masato Terashima, Takeshi Yoshida, Kiyohiro Sakai, Yoichi Otake, Atsuko Koyama, Kazuto Nishio, Kazuhiko Nakagawa

**Affiliations:** 1 Palliative Care Center, Cancer Center, Kindai University Hospital, 377-2 Ohno-higashi, Osakasayama City, Osaka, 589-8511 Japan; 2 Department of Psychosomatic Medicine, Kindai University Faculty of Medicine, 377-2 Ohno-higashi, Osakasayama City, Osaka, 589-8511 Japan; 3 Department of Medical Oncology, Kindai University Faculty of Medicine, 377-2 Ohno-higashi, Osakasayama City, Osaka, 589-8511 Japan; 4 Clinical Research Center, Kindai University Hospital, 377-2 Ohno-higashi, Osakasayama City, Osaka, 589-8511 Japan; 5 Department of Genome Biology, Kindai University Faculty of Medicine, 377-2 Ohno-higashi, Osakasayama City, Osaka, 589-8511 Japan; 6 Department of General Internal Medicine, Sakai City Medical Center, 377-2 Ohno-higashi, Osakasayama City, Osaka, 589-8511 Japan

**Keywords:** Opioid, Biomarker, Cancer pain, Randomized controlled trial

## Abstract

**Background:**

Cancer patients experience pain that has physiological, sensory, affective, cognitive, behavioral, and sociocultural dimensions. Opioids are used in treatment of pain in patients with various types of cancer. We previously showed that the catechol-O-methyltransferase (COMT) genotype is related to the plasma level of morphine and the required dose of morphine in an exploratory prospective study. The findings showed that a group of patients with a GG single nucleotide polymorphism (SNP) rs4680 in COMT required a significantly higher dose of morphine than a non-GG group. A biomarker for selection of opioids for cancer pain relief would be particularly useful clinically, and therefore we have planned a randomized comparative study of morphine and oxycodone, using the COMT rs4680 SNP as a biomarker. This study is aimed at verifying the assumption that patients in the GG group require an increased morphine dose for pain relief.

**Methods:**

The RELIEF study is a randomized, multi-institutional, open-label trial with a primary endpoint of the proportion of subjects requiring high-dose opioids, as calculated from the dose of a rescue preparation administered on day 0. Secondary endpoints include the Hospital Anxiety and Depression Scale, Short form McGill Pain Questionnaire-2, European Organization for Research and Treatment of Cancer QLQ-C15-PAL, Pain Catastrophizing Scale, and adverse events, Eligibility criteria are patients with advanced carcinoma with non-daily use of opioids in initial screening for registration; and cancer pain targeted for daily opioid treatment, NSAIDs or acetaminophen, NRS ≥3(average over 24 h), opioid-treatment naive within 30 h, no chemotherapy, radiotherapy, or bisphosphonate administration newly started within 2 weeks, and written informed consent at the time of second registration. Between November 2014 and June 2017, an estimated 110 patients from two sites in Japan were randomized (1:1) to morphine or oxycodone in GG and non-GG groups.

**Discussion:**

A method for selection of appropriate opioids in cancer patients is a high unmet medical need. This study was designed to evaluate the efficacy of different opioids in patients with cancer based on gene polymorphism, as the first potential multi-institutional registration trial to be conducted in cancer patients with pain.

**Trial registration:**

UMIN000015579 Date of registration: 4 November 2014. It is updated once every six months, the latest update is 30 June 2017.

Trial status.

The enrollment started in November 2014. At the time of manuscript submission (July 2017), Three-quarters of patients have participated. We thus expect to complete the recruitment by March 2018.

## Background

Opioids are important drugs for cancer pain relief, and definition of an appropriate required dose is needed to provide quick and potent pain relief. The required opioid dose is well known to vary widely among patients, but there have been few studies of biomarkers for the required dose and therapeutic efficacy, or for monitoring of pharmacodynamic effects of opioids. We have previously shown a relationship between cytokines and therapeutic efficacy of morphine [1] and a relationship of catechol-O-methyltransferase (COMT) gene polymorphism with the efficacy and dosage of morphine [2]. These findings emerged in an exploratory prospective study in the Cancer Clinical Research Program funded by a Health and Labor Sciences Research Grant from 2010 to 2012. In particular, we found that patients with a GG single nucleotide polymorphism (SNP) rs4680 in COMT require a significantly higher dose of morphine compared to non-GG patients.

A previous report indicated no relationship between genetic polymorphism and opioid requirement [3], but this report had several limitations, including analysis only in Western patients and uncertainty regarding control subjects, types of pain, use of concomitant medication, presence of symptoms other than pain, and lack of evaluation of pain associated with psychosocial background. Several other studies [4–6] have shown similar results to our preliminary findings, but a recent prospective study showed no relationship between oxycodone dosage and genetic polymorphism [7]. Compared to oxycodone, morphine is available in a greater number of dosage forms, has been used more widely, and is recommended as the first-line drug in many guidelines for cancer pain relief. There is also more information on dyspnea as an adverse effect of morphine. Furthermore, immediate-release morphine reaches a high serum level rapidly and has a shorter sustained duration, and thus is readily used as a rescue drug.

The rationale for the planned study is that morphine and oxycodone are common drugs used for cancer pain, but biomarkers for selection of these drugs, definition of the required dose, and therapeutic efficacy are not available. Based on the above background, there is clearly a practical clinical need for a biomarker in opioid therapy. To identify a biomarker for selection of opioids for cancer pain relief, we planned a randomized comparative study of morphine and oxycodone using the COMT rs4680 SNP as a biomarker. In accordance with criteria described in a previous study of pain [8], the subjects will be patients with a Numerical Rating Scale (NRS) score ≥ 3 averaged over 24 h. Parallel group comparison will be used for randomization in the study design, so as to generate high-quality evidence.

## Methods/design

### Aim, design and setting

**Fig. 1:**
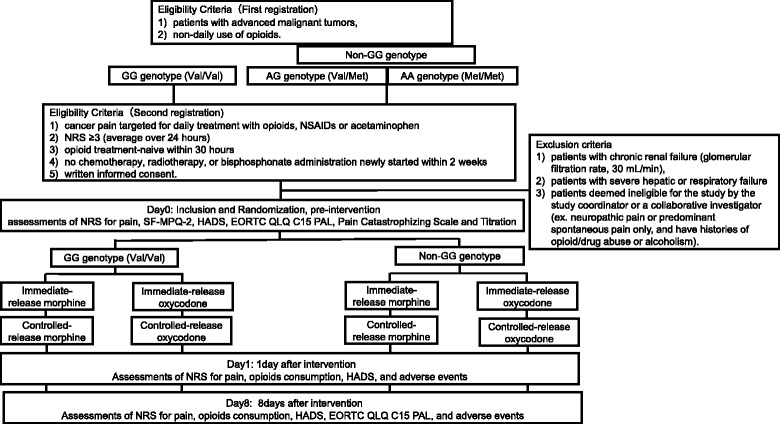
Design of the study

*Study objectives:* A randomized controlled trial (RCT) will be performed with the design shown in Fig. 1. The proportion of subjects requiring high-dose opioids (≥60 mg/day of morphine or ≥40 mg/day of oxycodone administered on day 0 will be calculated from use of immediate-release preparations and compared between the morphine and oxycodone groups using the morphine-equivalent dose in patients with the GG or non-GG COMT rs4680 SNP.

### Participants

Inclusion criteria are 1) patients with advanced malignant tumors, and 2) non-daily use of opioids. At the second registration, the criteria are 1) cancer pain targeted for daily treatment with opioids, NSAIDs or acetaminophen, 2) NRS ≥3 (average over 24 h), 3) opioid treatment-naive within 30 h, 4) no chemotherapy, radiotherapy, or bisphosphonate administration newly started within 2 weeks, and 5) written informed consent.

The exclusion criteria are 1) patients with chronic renal failure (glomerular filtration rate, 30 mL/min), 2) patients with severe hepatic or respiratory failure, and 3) patients deemed ineligible for the study by the study coordinator or a collaborative investigator (ex. neuropathic pain or predominant spontaneous pain only, and have histories of opioid/drug abuse or alcoholism).

### Endpoints

#### Primary endpoint

The primary endpoint is the proportion of subjects requiring high-dose opioids calculated from use of the immediate-release preparation on day 0 in a parallel group comparison.

#### Secondary endpoints

The secondary endpoints are the Hospital Anxiety and Depression Scale (HADS) score for anxiety and depression; the European Organization for Research and Treatment of Cancer (EORTC) QLQ-C15-PAL for score for QOL; the Short-Form McGill Pain Questionnaire 2 (SF-MPQ2) score for pain characterization; and the Pain Catastrophizing Scale (PCS) for estimation of impact on pain prognosis. Adverse events (e.g., constipation, somnolence, nausea, pruritus, ischuria) will be evaluated using the Common Terminology Criteria for Adverse Events (CTCAE) ver. 4.0.

### Measurement tools

#### Performance status (PS)

The European Cooperative Oncology Group (ECOG) PS system will be used for evaluation of PS by primary physicians [9].

#### Numerical rating scale (NRS)

The NRS will be used to evaluate pain for its better validity, sensitivity, and convenience compared to other scales [10] and its widespread use in many clinical studies.

#### Hospital anxiety and depression scale (HADS)

The HADS will be used for measurement of psychiatric symptoms (anxiety and depression) of patients with a physical disease. HADS is a screening tool that allows assessment based on a small number of items. Its reliability and validity have been verified internationally [11, 12].

#### European Organization for Research and Treatment of cancer (EORTC) QLQ-C15-PAL

EORTC QLQ-C15-PAL will be used for evaluation of patient QOL. The reliability and validity of the Japanese version have been confirmed [13].

#### Short-form McGill pain questionnaire 2 (SF-MPQ-2, Japanese version)

The SF-MPQ-2 will be used to examine differences in effects due to pain mechanisms. The reliability and validity of the Japanese version have been verified [14].

#### Pain catastrophizing scale (PCS)

The severity of cancer-related pain is influenced by engagement of patients in catastrophic thinking, such as "my pain will undoubtedly never improve" [15]. This effect will be measured using the Japanese version of the PCS, for which the validity and reliability have been shown [16].

#### Common terminology criteria for adverse events (CTCAE)

The worst grade of an adverse event during the preceding period will be assessed using the CTCAE v.4.0, Japanese Clinical Oncology Group (JCOG) version.

### Protocol treatment

**Fig. 2:**
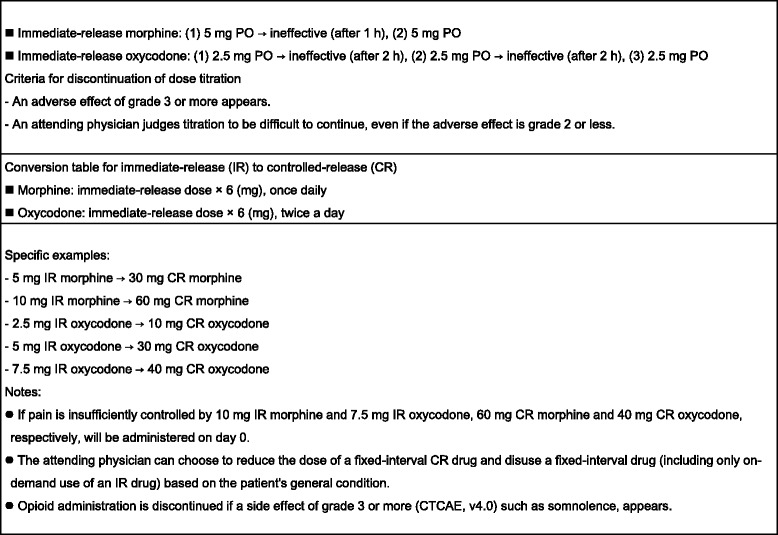
Methods for dose titration

In this prospective clinical study, cancer patients will undergo initial registration and genotyping for SNPs with a Taqman SNP Genotyping Assay (Life Technologies). In the first application of opioid treatment after occurrence of cancer pain, the patients will be divided into a GG group and a non-GG group based on the COMT rs4680 SNP and then undergo second registration, after which the protocol treatment will be started. Each group will be randomized into subgroups that will receive immediate-release morphine (T_max_ about 1 h) and immediate-release oxycodone (T_max_ about 2 h), respectively, with doses subjected to titration. Dose titration will be performed to decrease pain by ≥33% on the NRS pain scale, as well as reducing NRS to ≤3. The patients were tested with opioid according to the guideline for titration and following regular dosing (NCCN Guidelines™, Adult Cancer Pain) by specialized palliative care doctors. On this step, they explained potential benefits and adverse effects to their patients. Subsequently, the controlled-release opioid will be administered (Fig. 2). In all subgroups, the incidences of subjects requiring a high opioid dose (as a morphine-equivalent dose), psychological tests, and evaluation of QOL are defined as quantitative clinical endpoints to investigate the efficacies of morphine and oxycodone in the GG and non-GG groups. Candidate biomarkers related to onset of adverse effects of opioids will also be measured to examine correlations in an integrated manner. NRS, psychological tests, QOL evaluation, and blood collection will be performed before opioid treatment and on days 1 and 8 after starting treatment. The screening of biomarkers correlated with opioid adverse effects is performed as a subsidiary study. Randomization will be performed on a web page produced by To Field Inc. using the minimization method with modulating factors of age, sex, performance status (PS), and site of pain.

*Completion of treatment and use of other drugs*: Treatment is completed 8 days after the beginning of the protocol. As supportive therapy, the opioid dose can be increased or reduced as appropriate, based on the decision of a primary physician. Regarding combination therapy, there is no limitation on the use of any other drug.

*Subsidiary biomarker study*: This study will include screening for biomarkers correlated with adverse effects of opioids. Therefore, the following items will be examined as potential pharmacological biomarkers: (1) serum chemokine levels, (2) polymorphisms in opioid function-related genes, (3) serum glycan analysis, and (4) psychological tests, QOL scale and others. Treatment will be performed in accordance with normal guidelines and will not be changed for this study.

*Sample handling*: Blood will be collected at initial registration, before starting opioid treatment after second registration, and on days 1 and 8 after starting treatment. Samples will be collected and stored in the laboratory of the Department of Medical Oncology, Kindai University Faculty of Medicine. Samples will be encoded with identification numbers by a manager of personal information at the time of registration.

*Ethical issues*: All patients are required to provide written informed consent. The study will be performed in accordance with the Declaration of Helsinki and the International Conference on Harmonization and Good Clinical Practice. The protocol has been approved by the Institutional Review Board at each study site.

### Statistical analysis

The null hypothesis is that the proportion of subjects requiring high-dose opioids is equal between the morphine and oxycodone groups for subjects with the GG genotype. This null hypothesis will be evaluated using Fisher’s exact test at the one-tailed significance level of 2.5%. The 95% confidence interval (CI) of the difference in the proportion of subjects requiring high-dose opioids will be calculated as an estimate of the therapeutic efficacy. The proportion of subjects requiring high-dose opioids and the 95% CI will also be calculated in each group.

*Sample size calculation*: Our preliminary study [2] indicated that high-dose morphine was required by 36.8% of GG genotype subjects, and results for 100 subjects in a preceding study at Kindai University and 160 subjects in the series in this study suggested that this proportion was about 46%. On the basis of previous data and discussions at a conference at Kindai University Hospital, about 5% of these subjects are likely to require high-dose oxycodone. Thus, we assumed that 46% of subjects would require high-dose morphine and 5% would require high-dose oxycodone in the GG group. Under these assumptions, when we set the one-tailed significance level of 2.5% and power of 80%, Fisher’s exact test requires 31 GG subjects in each drug group; that is, 62 subjects with the GG genotype. Since the GG genotype is estimated to account for around 46% of the population, the number of registrations required is 135 subjects. Therefore, the target is defined as 140 subjects to allow for dropout and subjects who cannot be analyzed.

## Discussion

This study is the first multicenter RCT of the efficacy of opioids for cancer pain, other than psychogenic pain. We faced three major issues: (i) the heterogeneity of causes of pain, (ii) the absence of a standard of care in this setting, and (iii) the choice of the primary endpoint. Heterogeneity of causes of pains was handled by defining the subjects as patients with any cancer pain except for psychogenic pain. Next, we defined morphine and oxycodone as the standard of care, since these are opioids that are currently used as initial treatment in opioid-naïve patients. We planned to standardize selection for each opioid, because no standard technique has been established for this purpose. Finally, the primary endpoint was defined as the intergroup difference in the number of subjects requiring high-dose opioids based on the COMT rs4680 SNP, with the goal of examining this biomarker for selection of opioids, on the basis of results from previous studies.

The GG COMT rs4680 polymorphism may emerge as a factor predicting therapeutic efficacy, a need for lower doses of oxycodone compared to morphine to exhibit efficacy, and fewer adverse events. Exploratory research may show that this biomarker can also predict side effects. Such findings will contribute to basic understanding of the pharmacological profile and therapeutic efficacy of opioids, as well as establish a practical clinical method for rapid pain relief.

## Trial status

The study protocol was approved by the institutional review board in September 2014. Recruitment started in November 2014 and is currently ongoing.

## Abbreviations

CTCAE: Common Terminology Criteria for Adverse Events
ECOG: European Cooperative Oncology Group
EORTC: European Organization for Research and Treatment
HADS: Hospital Anxiety and Depression Scale
JCOG: Japan Clinical Oncology Group
NCCN: National Comprehensive Cancer Network
NRS: Numerical Rating Scale
PCS: Pain Catastrophizing Scale
RCT: Randomized controlled trial
SF-MPQ: Short-Form McGill Pain Questionnaire
